# Whole-body vibration as a passive alternative to exercise after myocardial damage in middle-aged female rats: Effects on the heart, the brain, and behavior

**DOI:** 10.3389/fnagi.2023.1034474

**Published:** 2023-03-07

**Authors:** Kata Tóth, Tamás Oroszi, Csaba Nyakas, Eddy A. van der Zee, Regien G. Schoemaker

**Affiliations:** ^1^Department of Neurobiology, Groningen Institute for Evolutionary Life Sciences (GELIFES), University of Groningen, Groningen, Netherlands; ^2^Research Center for Molecular Exercise Science, Hungarian University of Sports Science, Budapest, Hungary; ^3^Behavioral Physiology Research Laboratory, Health Science Faculty, Semmelweis University, Budapest, Hungary; ^4^Department of Cardiology, University Medical Center Groningen, Groningen, Netherlands

**Keywords:** isoproterenol-induced cardiac damage, exercise, whole body vibration (WBV), depressive-like behavior, cognition, neuroinflammation, brain collagen

## Abstract

**Background:**

Females with cardiovascular disease seem more vulnerable to develop concomitant mental problems, such as depression and cognitive decline. Although exercise is shown beneficial in cardiovascular disease as well as in mental functions, these patients may be incapable or unmotivated to perform exercise. Whole body vibration (WBV) could provide a passive alternative to exercise. Aim of the present study was to compare WBV to exercise after isoproterenol (ISO)-induced myocardial damage in female rats, regarding effects on heart, brain and behavior.

**Methods:**

One week after ISO (70 mg/kg s.c., on 2 consecutive days) or saline injections, 12 months old female rats were assigned to WBV (10 minutes daily), treadmill running (30 minutes daily) or pseudo intervention for 5 weeks. During the last 10 days, behavioral tests were performed regarding depressive-like behavior, cognitive function, and motor performance. Rats were sacrificed, brains and hearts were dissected for (immuno)histochemistry.

**Results:**

Significant ISO-induced cardiac collagen deposition (0.67 ± 0.10 vs 0.18 ± 0.03%) was absent after running (0.45 ± 0.26 vs 0.46 ± 0.08%), but not after WBV (0.83 ± 0.12 vs 0.41 ± 0.05%). However, WBV as well as running significantly reduced hippocampal (CA3) collagen content in ISO-treated rats. Significant regional differences in hippocampal microglia activity and brain derived neurotrophic factor (BDNF) expression were observed. Significant ISO-induced CA1 microglia activation was reduced after WBV as well as running, while opposite effects were observed in the CA3; significant reduction after ISO that was restored by WBV and running. Both WBV and running reversed the ISO-induced increased BDNF expression in the CA1, Dentate gyrus and Hilus, but not in the CA3 area. Whereas running had no significant effect on behavior in the ISO-treated rats, WBV may be associated with short-term spatial memory in the novel location recognition test.

**Conclusion:**

Although the female rats did not show the anticipated depressive-like behavior or cognitive decline after ISO, our data indicated regional effects on neuroinflammation and BDNF expression in the hippocampus, that were merely normalized by both WBV and exercise. Therefore, apart from the potential concern about the lack of cardiac collagen reduction, WBV may provide a relevant alternative for physical exercise.

## 1. Introduction

Many patients with cardiovascular disease may also experience mental disorders, including depression and cognitive impairment. These mental disorders are often overlooked or regarded as “natural” responses to a life-threatening condition. However, mental disorders can be associated with increased morbidity and mortality (Gharacholou et al., 2011; Meijer et al., 2013). Moreover, female patients seemed more vulnerable to developing heart failure-associated depression (Gottlieb et al., 2004; Eastwood et al., 2012) and cognitive decline (Ghanbari et al., 2013) than male patients. Although the worsening of cardiovascular prognosis by comorbid depression is well recognized (Nabi et al., 2010), anti-depressant treatment may alleviate depressive symptoms but does not improve cardiovascular prognosis (Thombs et al., 2008). In female patients, it may even deteriorate cardiovascular prognosis (Krantz et al., 2009). A rationale for therapy of this comorbidity is hampered by the lack of understanding of the heart–brain interaction and the potential difference in male and female patients.

Extensive evidence points to a role of a derailed (neuro)inflammatory response to cardiac damage, as a mechanism underlying the cardiovascular disease-depression association (Liu et al., 2013; Angermann and Ertl, 2018). However, the efficacy of anti-inflammatory therapy (Kosmas et al., 2019) seems rather poor. A lot of knowledge about the heart–brain interaction comes from animal studies. Heart failure induced by coronary artery ligation was associated with cognitive impairment (Hovens et al., 2016), as well as depressive-like behavior in rodents (Schoemaker and Smits, 1994; Wang et al., 2013; Frey et al., 2014). This behavior could be affected by cardiovascular-directed treatment (Schoemaker et al., 1996) and by treatment targeted at the brain and behavior (Grippo et al., 2003, 2006; Bah et al., 2011a,b; Ito et al., 2013). The isoproterenol (ISO)-induced cardiac damage model is often used as a way to induce focal cardiac damage and could be preferred above the coronary artery ligation model, as the former does not require brain-changing thoracic surgery (Hovens et al., 2016; Toth et al., 2021). In the ISO model, the effects of treatment on cardiovascular aspects are extensively studied (Nichtova et al., 2012; Ma et al., 2015; Alemasi et al., 2019), but studies on behavioral consequences are limited. Reduced exploratory behavior (Tkachenko et al., 2018) and declined sucrose preference after ISO (Hu et al., 2020) suggest depressive-like behavior, while the cognitive decline was also observed (Ravindran et al., 2020). Concomitant cardio and neuroprotective effects have been obtained in this model with Corvitin (Tkachenko et al., 2018), sodium thiosulfate (Ravindran et al., 2020), and traditional Chinese medicine Kai-Xin-San (Hu et al., 2020). However, these effects were only studied in young male rats, leaving potentially different effects in female rats unrevealed. Sex dimorphism in the response to ISO was already recognized in the 70s (Wexler et al., 1974; Wexler and Greenberg, 1979), and supported by our group (Toth et al., 2022b).

Exercise is generally acknowledged for its beneficial effects on physical as well as mental conditions in health and disease (Pedersen and Saltin, 2015). Recently, we showed that exercise training after ISO-induced cardiac damage could reverse the anxiety-like behavior in male rats (Toth et al., 2021), but not in female rats (Toth et al., 2022a). Physical exercise before ISO prevented cardiac fibrosis and the upregulation of pro-inflammatory cytokines (Ma et al., 2015; Alemasi et al., 2019), but exercise after ISO seemed to deteriorate the cardiac damage (Azamian Jazi et al., 2017).

However, not all patients are capable and/or motivated to perform physical exercise. For them, a passive form of exercise, such as whole-body vibration, could provide an alternative (Runge et al., 2000; Zhang et al., 2014). Whole-body vibration (WBV) is a passive mechanical stimulation on a vibrating platform (van Heuvelen et al., 2021). In addition to increased muscle strength (Annino et al., 2017) and aerobic fitness (Zhang et al., 2014), WBV was associated with improved wound healing (Wano et al., 2021) and a reduced inflammatory phenotype (Weinheimer-Haus et al., 2014). Moreover, we recently showed that WBV improved motor performance, spatial memory, and anxiety-like behavior in aged rats (Oroszi et al., 2022a). However, these effects seemed more pronounced in male than in female rats. In a study of middle-aged female rats, treated with WBV from 1 to 30 days after mid-cerebral ischemia-reperfusion, decreased inflammasome activation (caspase-1 and IL1-beta) and increased brain-derived neurotrophic factor (BDNF) expression were observed, concomitant with infarct reduction (Raval et al., 2018).

Taken together, female patients with cardiovascular disease seemed more prone to develop mental disorders; beneficial effects of exercise training on behavior were observed in male rats with ISO-induced myocardial infarction (Toth et al., 2021), but not in female rats; WBV has indicated a passive alternative to exercise. Therefore, the present study aimed to explore WBV as an alternative to physical exercise in female rats after ISO-induced myocardial infarction, regarding its effects on the heart, the brain, and behavior.

## 2. Methods

### 2.1. Animals and experimental design

#### 2.1.1. Animals

Animals were housed in groups of two or three in cages of 30^*^42^*^20 cm with sawdust as bedding in the conventional animal facility of the University of Physical Education, Hungary, in a room with 22 ± 2°C and humidity of 50 ± 10%. The light was provided from 6 a.m. to 6 p.m. CEST. Experiments were performed approximately between 9 a.m. and 5 p.m. Standard rodent chow (LT/R, Innovo Ltd., Gödöllo, Hungary) and tap water were provided *ad libitum*. All methods were performed in accordance with the ARRIVE guidelines. Experimental animals and procedures were approved by the local animal committee of the University of Physical Education, Budapest, Hungary.

#### 2.1.2. Pilot

Exposure time–effect relation of WBV Before starting the main study on WBV as an alternative to exercise, a pilot exposure time–effect study was performed in order to find the optimal WBV exposure time per session for our female rats. For that, 28 female Wistar rats were collected from the breeding colony of the University of Sports Science, Hungary. Rats were randomly divided into four experimental groups, receiving 5 weeks (one time a day and 5 days a week) of treatment with either pseudo-WBV (0 min), or 5, 10, or 20 min of WBV per day. At the end of these 5 weeks, effects on behavior were evaluated, regarding open field (OF) exploration, short-term memory in the novel object/novel location recognition tests (NOR/NLR), and motor performance in the balance beam and grip hanging tests. For details about the WBV and behavioral testing, refer to Section 2 of the main study.

#### 2.1.3. Main study

**Figure 1:**
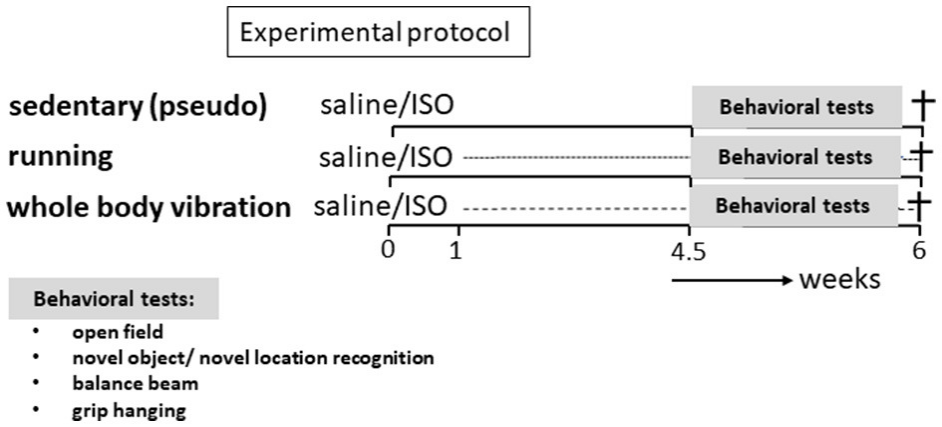
Experimental protocol. Rats (*n* = 64) were subdivided into six experimental groups. Part of the rats (*n* = 25) received two saline injections and the other part (*n* = 39) received two ISO injections, both 24 h apart. One week later, rats were subdivided into a sedentary group, receiving the pseudo-intervention, an exercise intervention group (treadmill running), and a whole-body vibration intervention group, for 5 weeks. In the last 10 days of this period, behavioral tests were performed, and rats were subsequently killed.

Sixty-four middle-aged (on average 12 months old) female Wistar rats were obtained from the breeding colony of the University of Sports Science, Hungary. Rats were randomized to six experimental groups: running ISO/saline, WBV ISO/saline, and as control pseudo-ISO/saline, and were subjected to the protocol presented in Figure 1. For that, rats were treated with ISO to induce heart lesions or received saline injections. After 1 week of recovery, rats were either subjected to 5 weeks of WBV or treadmill running or received pseudo-treatment (sedentary). Exploratory behavior, cognitive performance, and motor function were assessed during the last 10 days of the training period. After completion of all tests, animals were killed, and heart and brain tissues were collected for further analyses of cardiac collagen, brain collagen, neuroinflammation, and neuronal function. This experimental protocol is similar to the one described in detail in our previous studies (Toth et al., 2021; Oroszi et al., 2022a).

### 2.2. Interventions

#### 2.2.1. Cardiac damage

Acute cardiac damage was induced by isoproterenol hydrochloride (ISO; C_11_H_7_NO_3_·HCl: ISO), a non-selective β-adrenoceptor agonist that mimics the histological, physical, and endocrinological events of human myocardial infarction presumably by myocardial hyperactivity-induced ischemia and energy depletion (Wexler et al., 1968). Rats were injected subcutaneously with ISO (Sigma Aldrich) in a dose of 70 mg/kg dissolved in 1 ml/kg saline (Toth et al., 2021, 2022a,b). Control animals received 1 ml/kg saline. Both groups received two injections with 24 h in between, according to the protocol described by Ravindran et al. (2020).

#### 2.2.2. Whole-body vibration

Rats received a single vibration session of 10 min, five times per week, for 5 consecutive weeks, using a vibration platform (MarodyneLiV—low-intensity vibration; BTT 129 Health GmbH, Germany), as described in detail elsewhere (Oroszi et al., 2022a). The platform ensures constant vibration exposure with a frequency of 30 Hz and an amplitude of 50–200 microns. Rats were placed in empty individual cages on the platform (the same shape as the individual home cage, but without bedding). WBV took place in an adjacent room with the same climate conditions as the housing room.

#### 2.2.3. Treadmill running

Saline- and ISO-treated groups were assigned to a treadmill running protocol on a six-lane rat treadmill (Tartonik Elektronika, Italy) with individual lanes of 12^*^54^*^13 cm, as described in detail previously (Toth et al., 2021). The training program lasted for 5 weeks, five times per week on each weekday. On the 1st week of the training program, rats were habituated to running: on the 1st day, rats started with 10 min of running with a maximal speed of 10 m/min, which was gradually increased to 30 min and a maximal speed of 18 m/min (moderate intensity; ~65% of VO_2_max; Hoydal et al., 2007) by the 5th day. For the following 4 weeks, each running session lasted 30 min at 18 m/min.

#### 2.2.4. Pseudo-WBV/running

Rats that received pseudo-treatment served as sedentary controls for both WBV and exercise. Pseudo-treatment consisted of either 10 min on the turned-off vibrating platform or 30 min on the turned-off treadmill, on alternate days, for 5 weeks, 5 days a week.

### 2.3. Behavior

#### 2.3.1. General

Effects on behavior were tested during the last 10 days of the protocol (refer to Figure 1). Open field behavior (OF) was used to assess anxiety/depressive-like behavior (Schoemaker and Smits, 1994). Short-term memory was tested in the novel object recognition (NOR) and the novel location recognition test (NLR), as a measure for cognitive effects (Hovens et al., 2014b). Effects on motor performance were obtained in the balance beam test for motor coordination (Song et al., 2006) and in the grip strength test (Shear et al., 1998) for effects on muscle strength. Tests have been described in detail in our previous study (Oroszi et al., 2022a). All tests were recorded with a digital video camera (Canon Legria HFR106, Canon Inc., Tokyo, Japan) and stored on a memory card for later offline analyses.

#### 2.3.2. Open field

An open-field exploration test was performed to assess exploratory and anxiety-related behavior, as we described earlier (Toth et al., 2021). A round-shaped arena (diameter of 80 cm) was divided into an inner circle (diameter of 48 cm; center area) and an outer annulus (wall area). Initially, the area was divided into three concentric circles: wall, middle, and center (each 16 cm wide). However, as the rats hardly moved into the so-defined center area, this center area plus the middle area were taken together as “çenter” in our measurements. Animals were placed in the arena and allowed to explore for 5 min. After each animal, the arena was cleaned with 70% ethanol to remove smell cues. Time spent in the center and the wall area, as well as the number of visits to the center, were obtained using Eline software (University Groningen, the Netherlands). The total number of crossings between the initially defined three areas was used as an estimate for locomotion activity and exploration.

#### 2.3.3. Novel object and novel location recognition

To assess short-term visual memory, which depends primarily on prefrontal cortex function, a novel object recognition test (NOR) was performed, while short-term spatial memory, associated with hippocampal function, was assessed in the novel location recognition test (NLR), as described previously (Hovens et al., 2014b). After habituation to the test environment, the test battery consisted of three phases, each lasting 3 min, with 1 min in-between: For habituation, the animal was placed in the test box and allowed 3 min to explore the set-up; then the rat was presented two identical objects and allowed to explore those for 3 min. Subsequently, objects were removed and after 1 min placed back, but one of them in a different location than in the previous phase (NLR), followed by exploration for 3 min. Finally, after being removed for 1 min, the objects were presented again, but now one of the two identical objects was replaced by a different object and put in the same location as in the preceding phase (NOR). Between the phases, the objects were removed and cleaned with 70% ethanol to remove smell cues. After each animal, the test box and objects were also cleaned with 70% ethanol. Time spent exploring the objects was measured using Eline software (the University of Groningen, the Netherlands). Preference for the novel location or the novel object was calculated by dividing the time spent exploring the novel location or novel object by the time spent exploring both objects. Preference of 50% indicated chance level = no recognition. Results from rats that did not explore the objects or only one of them were excluded from further analyses.

#### 2.3.4. Balance beam test

A balance beam test was conducted two times on separate days to analyze motor coordination (Oroszi et al., 2022a). A 150-cm long and 2-cm wide wooden slat was positioned horizontally at 1 m above the floor, and on one end connected to the home cage of the rat, as a target. On the first day, the rats were trained to walk across the suspended beam, at which the rats performed three trials [50 cm, 100 cm, and one full test (150 cm)]. After these three training trials, the rats performed three full test trials. On the second day, the rats performed also full three test trials. The average latency to reach the home cage was used as a measure of performance on the balance beam. Animals who were unable and/or unwilling to perform the test procedure were excluded from the final statistical analysis.

#### 2.3.5. Grip strength test

The grip strength test was performed two times on separate days and included three trials per day (Oroszi et al., 2022a). Animals were held by their trunk and were guided to get grip on a suspended wood beam (2 mm in diameter, 35 cm in length, and 50 cm above the surface of the table) by their forepaws. Time until drop-off was recorded. During the three trials, the animals were rotated to offer time for recovery between the trials. The average of the six trials over the 2 days was utilized for statistical analysis.

### 2.4. Tissue collection and processing

At the end of the experiment, rats were anesthetized with 6% sodium pentobarbital solution and injected intraperitoneally (2 ml/kg). Rats were transcardially perfused with heparinized (1 ml/l) 0.9% saline until the liver turned pale. The right gastrocnemius and soleus muscles as well as the heart and the brain were dissected and weighed. The brain and the heart tissues were immersion fixated in 4% buffered formaldehyde freshly depolymerized with paraformaldehyde. After 4 days, tissues were washed in 0.01 M phosphate-buffered saline (PBS), dehydrated using a 30% sucrose solution, and subsequently quickly frozen in liquid nitrogen and stored at −80°C until 25 μm coronal sections were cut using a microtome. Heart sections and three brain sections were placed on glass immediately after cutting and processed for histochemical staining. The remaining brain sections were stored free-floating in 0.01 M (PBS) containing 0.1% sodium azide at 4°C till further processing for (immuno)histochemistry.

### 2.5. (Immuno)histochemistry

#### 2.5.1. Microglia

To visualize microglia, immunohistochemical staining of ionized calcium binding adaptor molecule 1 (IBA-1) was performed, as described in detail previously (Hovens et al., 2014a). In brief, after incubation for 3 days with 1:2,500 rabbit-anti IBA-1 (Wako, Neuss, Germany) in 2% bovine serum albumin, 0.1% triton X-100 at 4°C, followed by a 1 h incubation with 1:500 goat-anti-rabbit secondary antibody (Jackson, Wet Grove, USA) at room temperature, sections were then incubated for 2 h with avidin–biotin–peroxidase complex (Vectastain ABC kit, Vector, Burlingame, USA) at room temperature. Labeling was visualized by using a 0.075 mg/mL diaminobenzidine (DAB) solution activated with 0.1% H_2_O_2_. Sections were mounted onto glass slides and photographs were taken from the dorsal hippocampus (hippocampus) at 200 times magnification (Toth et al., 2022b). Microglia morphology was analyzed in the different areas of the dorsal hippocampus: CA1, CA3, dentate gyrus (DG), and hilus, according to our previous publication (Hovens et al., 2014a), regarding coverage, density, cell size, cell body area, and processes area. Microglia activity was calculated as cell body area/total cell size (Hovens et al., 2014a).

#### 2.5.2. Brain-derived neurotrophic factor

For brain function, brain slices were stained for brain-derived neurotrophic factor (BDNF), as described previously (Hovens et al., 2014b). In brief, sections were blocked for 1 h with 5% normal goat serum, then incubated with 1:1,000 rabbit-anti BDNF antibody (Alomone Labs, Israel) in 1% BSA, followed by incubation with 1:5,000 goat-antirabbit secondary antibody (Jackson, Wet Grove, USA). Photographs were taken at 50× magnification (Toth et al., 2022b) from the different areas of the dorsal hippocampus, CA1, CA3, DG, and hilus, and BDNF expression was obtained as corrected optical density (Image-J) compared to an underlying reference area (Hovens et al., 2014b).

#### 2.5.3. Collagen

In the heart, the percentage of collagen was used to measure cardiac damage. For that, 25 μm thick transverse slices at mid-ventricular and apex levels of the heart were stained with Sirius red (Sigma, Aldrich) and Fast green as counterstaining (Hovens et al., 2016). Color pictures were taken and enlarged to cover the complete left ventricle in the image analyses screen. Image analysis (Image Pro Plus, USA) was used to measure the collagen-positive (red) area and was expressed as a percentage of the total left ventricular tissue area. Since WBV may also affect collagen in other organs than the heart, a similar Sirius red/Fast green stain was performed on three brain sections that were immediately placed on glass, using the same procedure as was used for the cardiac sections.

Because of a positive collagen signal observed in the hippocampus in this pilot, for a subgroup of rats randomly selected from the experimental groups, free-floating sections were stained with Sirius red (without Fast green), after thoroughly washing (two times daily for 4–5 days) to remove the azide. Photographs were taken from the granular layers of the dorsal hippocampus (CA1, CA2, CA3, DG, and hilus; 100× magnification), and collagen expression was obtained as corrected optical density, compared to the underlying reference area (Image-J).

### 2.6. Data analyses

Data are presented as mean ± 95% confidence interval (CI; figures) and standard error of the mean (SEM; tables). Results more than two times the standard deviation of its group were considered outliers and were excluded before analyses (maximally one per experimental group). Results were compared using a two-way analysis of variance (ANOVA) with a least square difference (LSD) *post-hoc* test, with saline/ISO and sedentary/runner/whole-body vibration as factors. Association between selected parameters was measured with Pearson linear correlation. For the novel object/novel location recognition tests, outcomes were also tested against chance level (=50%), using a single sample *t*-test. A *p*-value of < 0.05 was considered statistically significant and is denoted as ^*^. Potentially relevant tendencies (*p* < 0.1) were mentioned as well.

## 3. Results

### 3.1. WBV exposure time–effect pilot

**Figure 2:**
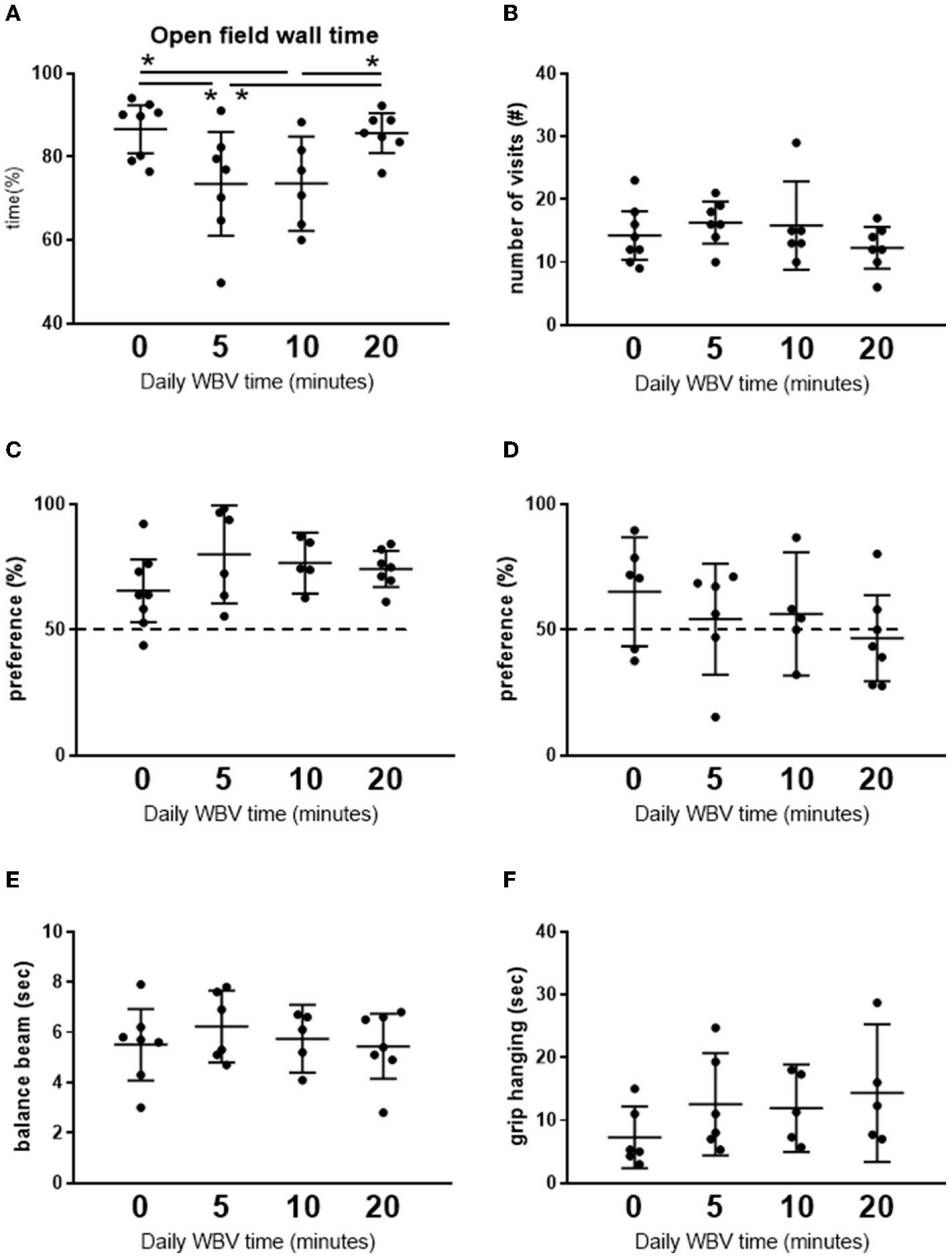
**(A, B)** Results from the open field test. **(C, D)** Short-term memory in the NOR and NLR test, respectively. **(E, F)** Effects on motor function. Results from the pilot exposure time-effect study on the different whole-body vibration (WBV) session times (*n* = 5–8 per group). Rats were subjected to 5 weeks daily (5 days a week) WBV for 0 (pseudo), 5, 10, or 20 min. Behavioral responses were obtained according to descriptions in the methods section. *Significant effect (*p* < 0.05).

To obtain insight into the effects of different exposure times of WBV in order to find an effective protocol to compare with the active exercise in our study, a pilot exposure time—effect study on behavior was performed. The results are presented in Figure 2. We tested daily for 5, 10, or 20 min against pseudo-treatment (=0 min). Five and ten minutes of WBV per day had similar effects, whereas the effects of 20 min per day appeared deviant. Daily 5 or 10 min of WBV significantly reduced anxiety-like behavior in the OF, without effects on short-term memory and balance beam performance, while muscle strength measured as grip hanging was slightly improved. From these results, we chose 10 min of WBV per day to use in our main study.

### 3.2. Main study

#### 3.2.1. General

Mortality in the ISO group was 26% (10 out of 39 rats), whereas none of the saline-treated rats died. At the start of the experiment, rats weighed on average 249 ± 3 g, with no differences between the experimental groups. Before killing, the rat's body weight was, on average, 251 ± 3 g. Body weights and organ weights corrected for body weight are presented in Table 1. Neither body weight nor relative organ weights were different between the experimental groups.

**Table 1:** Body weight (bw) and relative organ weights before killing in the different experimental groups.

	**Sed saline**	**Sed ISO**	**Run saline**	**Run ISO**	**WBV saline**	**WBV ISO**
*n*	8	9	8	10	9	10
Body weight (g)	253 ± 7	247 ± 4	258 ± 12	254 ± 7	250 ± 10	245 ± 7
Heart weight (%bw)	0.38 ± 0.02	0.40 ± 0.01	0.40 ± 0.02	0.39 ± 0.02	0.38 ± 0.01	0.40 ± 0.01
Brain weight (%bw)	0.78 ± 0.02	0.79 ± 0.02	0.75 ± 0.03	0.77 ± 0.02	0.79 ± 0.03	0.79 ± 0.01
Soleus weight (%bw)	0.084 ± 0.004	0.081 ± 0.003	0.080 ± 0.003	0.076 ± 0.003	0.081 ± 0.002	0.083 ± 0.005
Right gastronemicus weight (%bw)	0.58 ± 0.01	0.60 ± 0.01	0.59 ± 0.02	0.60 ± 0.01	0.60 ± 0.01	0.58 ± 0.02

Sed, sedentary; ISO, isoproterenol; Run, runner; WBV, whole-body vibration.

No significant effects of ISO vs. saline, nor interventions were observed.

#### 3.2.2. Cardiac collagen

**Figure 3:**
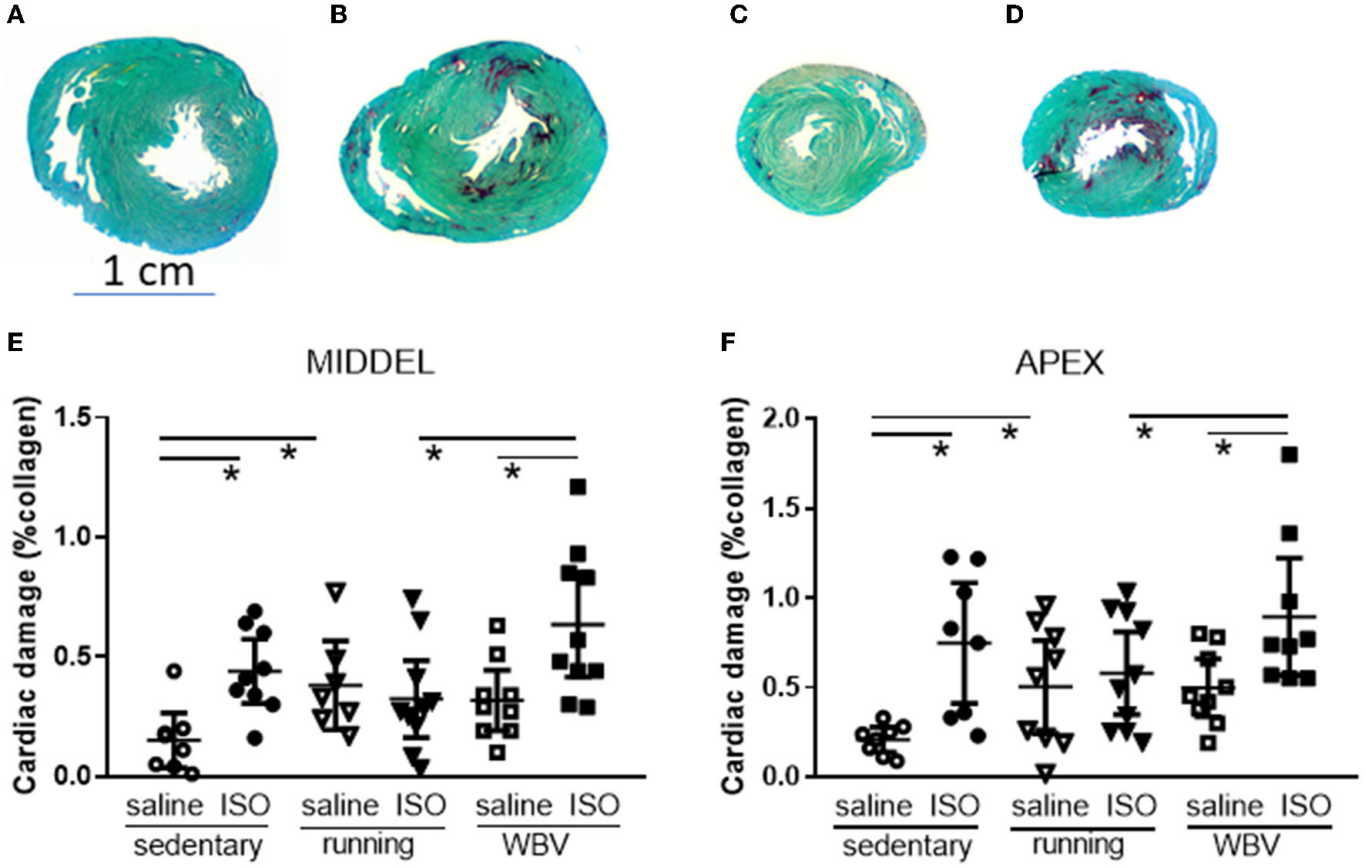
Presentation of midventricular and apex sections stained for Sirius red (red) and Fast green (green), from sedentary saline [**(A, C)**, respectively] and isoproterenol whole-body vibration-treated rats [**(B, D)**, respectively], showing increased Sirius red positive areas after ISO treatment. Lower panels: actual measurements of the percentage of collagen in the left ventricle as Sirius Red positive area at mid-ventricular **(E)** and apex level **(F)**, in saline or isoproterenol (ISO)-treated rats, under sedentary conditions, or after 5 weeks of exercise training (running) or whole-body vibration (vibration). *Significant difference between indicated groups (*p* < 0.05).

The percentage of collagen in the left ventricle measured at mid-ventricular and apex levels was used as a measure of cardiac damage (Figure 3). Two-way ANOVA revealed a significantly elevated collagen percentage after ISO compared to saline-treated rats at both ventricular levels (middle: *p* = 0.003; apex: *p* = 0.002), and a significant intervention effect (*p* = 0.040) and interaction effect (*p* = 0.024) only at the mid-ventricular level. *Post-hoc* analyses indicated that the fibrotic effect of ISO, seen in the sedentary rats (middle: *p* = 0.008; apex: *p* = 0.001), was absent after running, but not after WBV. In fact, WBV in ISO rats tended to even further increase collagen (mid-ventricular level; *p* = 0.052). However, the effect of running may at least in part be attributed to increased collagen in the control saline-treated rats, since running (*p* = 0.030), and to a lower extent WBV (ns), by itself already increased cardiac collagen.

#### 3.2.3. Hippocampal collagen

**Figure 4:**
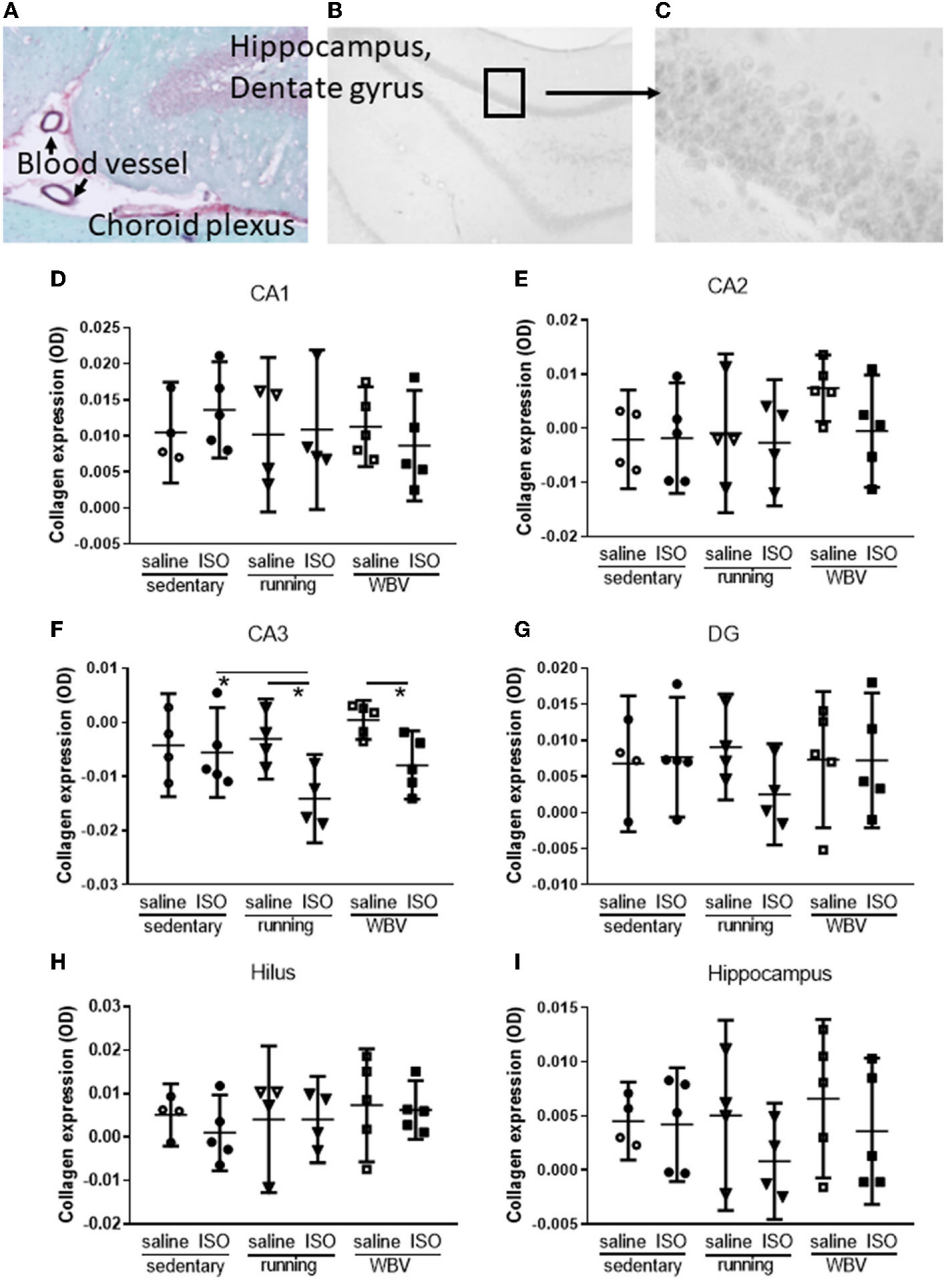
**(A)** Example of hippocampal dentate gyrus stained with Sirius red (red) and Fast green (green), showing positive staining for collagen (red) in blood vessel walls, fibrous tissue of the choroid plexus, and the granular layer of the dentate gyrus, indicating the presence of collagen in brain tissue (50× magnification). **(B)** A typical example of a black and white photograph of a section stained with Sirius red only (magnification 20×). **(C)** Detail of **(B)** as was used to quantify gray values (100 × magnification). **(D–H)** Actual measurements of optical density in the different areas of the hippocampus, as well as for the whole hippocampus **(I)** in saline- or isoproterenol (ISO)-treated rats, under sedentary conditions (*n* = 4 and *n* = 5, respectively), or after 5 weeks of exercise training (running; *n* = 4 each) or whole-body vibration (vibration; *n* = 5 each). *Significant difference between indicated groups (*p* < 0.05).

Figure 4A shows photographs of the dorsal hippocampus (dentate gyrus), stained according to the same protocol as had been used for cardiac sections; Sirius red/Fast green. The photograph showed that in addition to the expected positive staining (red) of blood vessel walls and the fibrous tissue of the choroid plexus, the granular layers (containing mostly the neuronal soma) in the hippocampus also stained positive for collagen. The Sirius red/Fast green staining protocol for sections collected directly on the glass, however, could not be used for free-floating sored sections, potentially due to the presence of azide in the storage solution. Therefore, in order to visualize collagen in the hippocampus in the sections from our experimental groups, 4–5 days of daily washing appeared to be necessary to obtain a sufficient signal-to-noise ratio, and quantification was obtained from optical density (Figures 4B, C). The results of the measurement were shown in the lower part of the figure. While the hippocampus (Figure 4I) overall did not show statistically significant differences between the experimental groups regarding collagen, effects seemed to differ locally. In the CA1 (Figure 4D), DG (Figure 4G), and hilus areas (Figure 3H), no differences were observed between groups. However, in the CA2 area (Figure 4E), the saline-treated WBV rats appeared to show consistently increased collagen expression, whereas, in all other groups, virtually no expression was observed. Although this did not result in significant differences between groups in the CA2, differences reached statistical significance (*p* = 0.011) in the CA3 area (Figure 4F). Surprisingly, running (*p* = 0.007) as well as WBV (*p* = 0.019) caused a significant decline of collagen in ISO-treated, compared to saline-treated, rats. Moreover, running after ISO significantly declined collagen expression (*p* = 0.023) compared to sedentary ISO rats.

#### 3.2.4. Hippocampal microglia activity and BDNF expression

**Figure 5:**
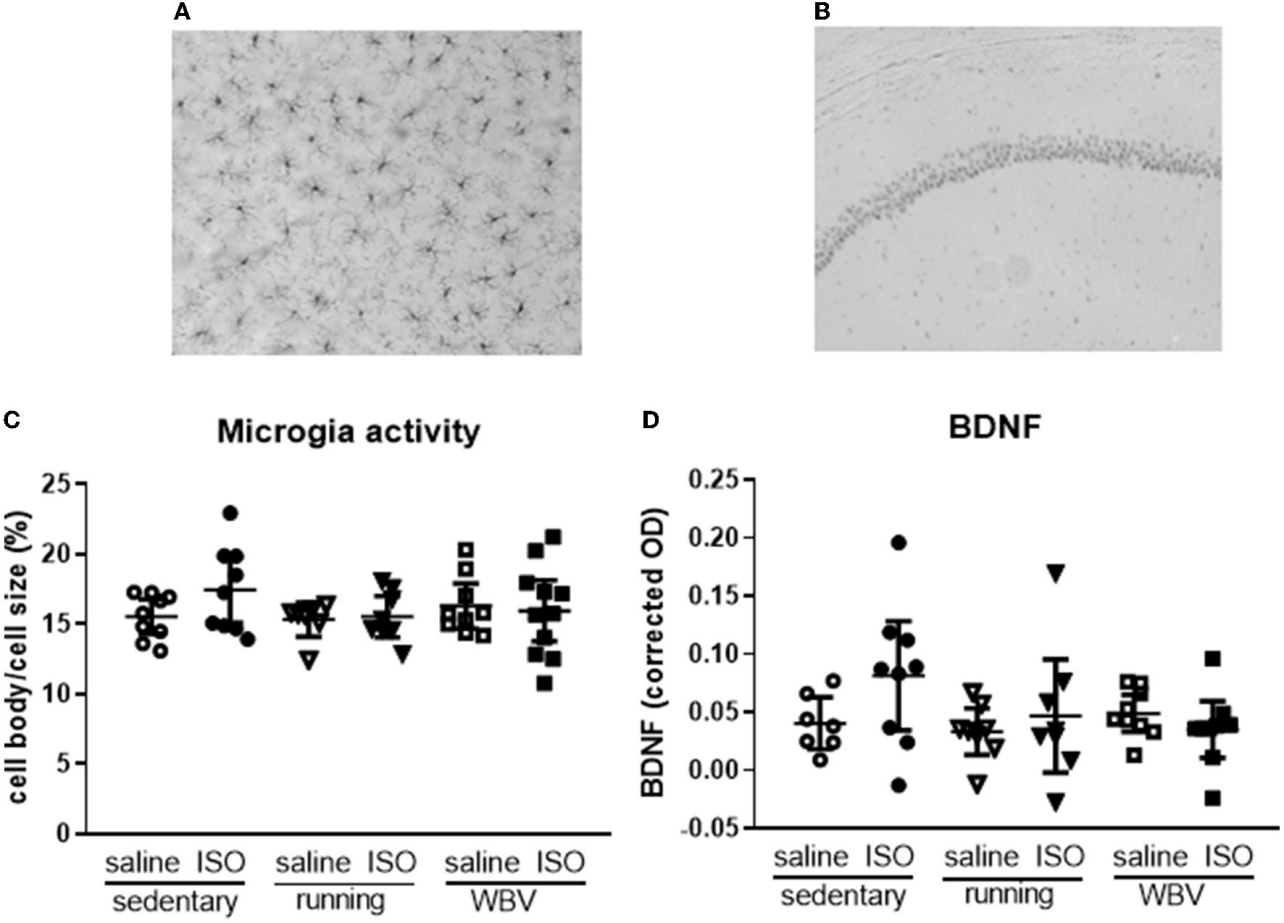
Typical pictures of IBA-1—**(A)** (200× magnification) and BDNF staining **(B)** (50× magnification) from the CA1 region of the hippocampus. Mean hippocampal microglia activity **(C)** and BDNF expression **(D)** in saline- or isoproterenol (ISO)-treated rats, under sedentary conditions (*n* = 8 and *n* = 9, respectively), or after 5 weeks of exercise training (running; *n* = 6–8 and *n* = 8, respectively) or whole-body vibration (vibration; *n* = 8–9 and *n* = 9, respectively).

**Figure 6:**
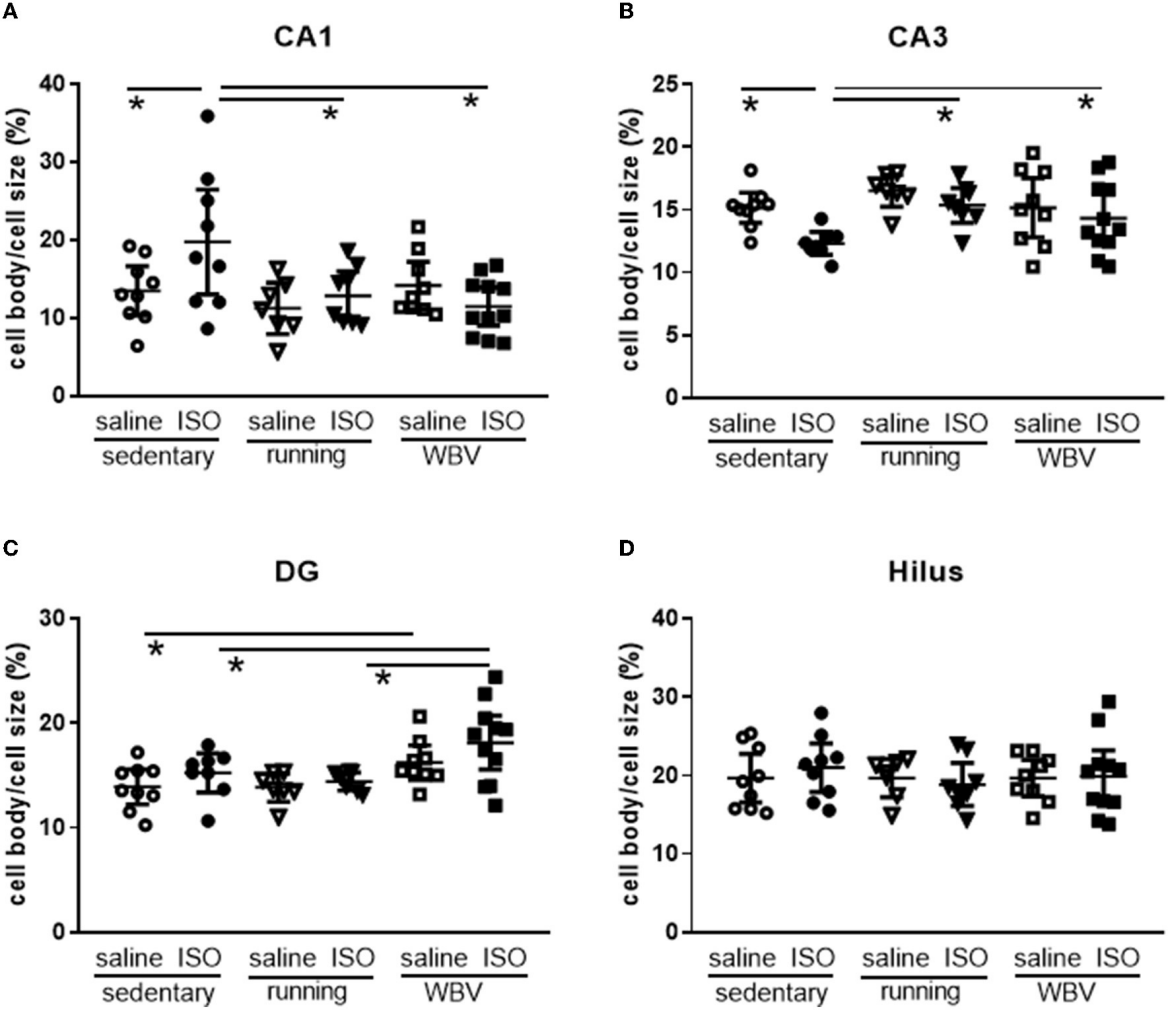
**(A)** CA1, **(B)** CA3, **(C)** DG, and **(D)** Hilus. Measurement of microglia activity as cell body/cell size in the different areas of the hippocampus in saline- or isoproterenol (ISO)-treated rats, under sedentary conditions (*n* = 8 and *n* = 9, respectively), or after 5 weeks of exercise training (running; *n* = 6 and *n* = 8, respectively) or whole-body vibration (vibration; *n* = 8 and *n* = 10, respectively). *Significant difference between indicated groups (*p* < 0.05).

**Figure 7:**
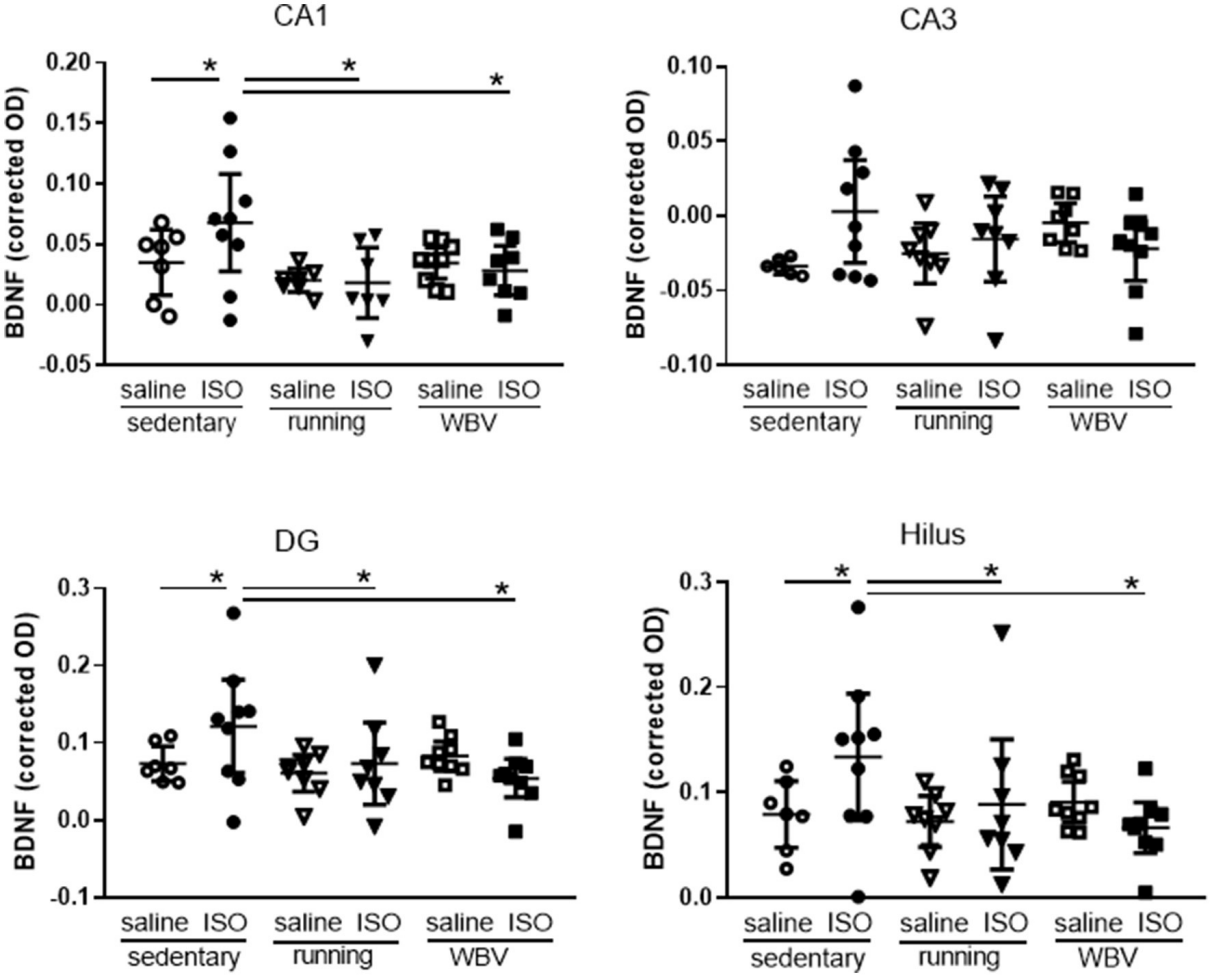
Expression of brain-derived neurotrophic factor (BDNF) in the different areas of the hippocampus in saline- or isoproterenol (ISO)-treated rats, under sedentary conditions (*n* = 7 and *n* =9, respectively), or after 5 weeks of exercise training (running; *n* = 8 each) or whole-body vibration (vibration; *n* = 9 each). *Significant difference between indicated groups in *post-hoc* analyses (*p* < 0.05).

Effects on neuroinflammation were obtained from morphological changes in microglia represented by increased cell body-to-cell size ratio and microglia activity (Figures 5A, C). No significant differences due to either ISO treatment or running/WBV interventions were observed in the overall hippocampal microglia activity score. Similarly, no effects of ISO or intervention were observed on average hippocampal BDNF expression (Figure 5D). However, within the hippocampus, the different regions responded differently as described in Sections 3.2.5 and 3.2.6, respectively, and illustrated in Figures 6, 7.

#### 3.2.5. Hippocampal neuroinflammation

Analyzing the different areas within the hippocampus (Figure 6), two-way ANOVA revealed significant differences between groups in the CA1 (*p* = 0.010), CA3 (*p* = 0.013), and DG (*p* = 0.003), but not in the hilus area. In the CA1 (*p* = 0.022), CA3 (*p* = 0.011), and DG (*p* = 0.001), differences could be attributed to a significant effect of the intervention (sedentary, running, or WBV); in the CA3 area, this effect appeared on top of a significant ISO/saline effect (*p* = 0.023). *Post-hoc* testing revealed a significant increase in microglia activity due to ISO in the CA1 (*p* = 0.011), no effect in the DG, and a significant decline in activity in the CA3 (*p* = 0.010). In both the CA1 and CA3 areas, effects of ISO were normalized by WBV as well as running exercise (CA1: *p* = 0.001 and *p* = 0.007, for WBV and running exercise, respectively; CA3: *p* = 0.050 and *p* = 0.007, for WBV and running exercise, respectively). In the DG, WBV seemed to activate microglia in saline (*p* = 0.050) as well as ISO-treated rats (*p* = 0.014). Since the microglia activity (cell body to total cell size ratio) resulted from a calculation based on measured morphological features, effects on the underlying parameters are presented in Table 2. The opposite responses of the CA1 and CA3 microglia activity to ISO, as described in Figure 6, seemed reflected in the underlying morphological parameters. In the CA1, total cell size declined after ISO, due to loss of processes area with a (ns) increase in cell body size, while running, and to a lesser extent WBV, increased cell size by increasing processes size. In the CA3, cell size as well as processes tended to increase (*p* < 0.1), while cell bodies declined in size, resulting in reduced microglia activity after ISO in this area. WBV and exercise after ISO seemed to normalize these parameters. The DG and hilus microglia were not so much altered after ISO. WBV, but not exercise, seemed to reduce cell size and process size, resulting in higher microglia activity (Figure 6).

**Table 2:** Morphological parameters of microglia obtained in different hippocampal areas: CA1, CA3, dentate gyrus (DG), and hilus.

**Experimental group/hippocampal area**	**Sedentary saline**	**Sedentary ISO**	**Runner saline**	**Runner ISO**	**Vibration saline**	**Vibration ISO**
*n*	8	9	6	8	8	10
**CA1**						
Density (#/area)	63.6 ± 3.1	57.9 ± 4.1	65.0 ± 3.5	67.4 ± 5.4	58.1 ± 5.3	55.6 ± 4.2
Coverage (%)	4.4 ± 0.5	3.4 ± 0.2	5.1 ± 0.6	3.9 ± 0.3	4.2 ± 0.6	3.5 ± 0.3
Cell size (px)	760 ± 59	**605 ± 41** ^**#**^	**899 ± 81** ^*^	**702 ± 35** ^**#**^	**717 ± 28** ^**+**^	686 ± 24
Cell body size (px)	88 ± 7	97 ± 9	86 ± 4	78 ± 6	88 ± 6	**68 ± 5** ^* #^
Processes size (px)	685 ± 61	**530 ± 42** ^**#**^	822 ± 85	**630 ± 35** ^**#**^	**636 ± 27** ^**+**^	607 ± 25
**CA3**						
Density (#/area)	68.9 ± 3.0	69.2 ± 4.1	**56.3 ± 3.2** ^*^	**67.3 ± 2.7** ^**#**^	59.7 ± 3.0	**59.0 ± 3.6** ^*^
Coverage (%)	3.7 ± 0.2	3.8 ± 0.3	3.2 ± 0.2	**4.0 ± 0.2** ^**#**^	3.2 ± 0.2	**3.7 ± 0.2** ^**#**^
Cell size (px)	629 ± 16	688 ± 28	596 ± 23	**592 ± 23** ^*^	627 ± 26	**570 ± 17** ^*^
Cell body size (px)	95 ± 4	**82 ± 2** ^**#**^	98 ± 4	91 ± 4	93 ± 4	**81 ± 4** ^**#**^
Processes size (px)	542 ± 15	602 ± 23	516 ± 23	**523 ± 21** ^*^	541 ± 29	**513 ± 19** ^*^
**DG**						
Density (#/area)	70.3 ± 3.5	68.8 ± 4.9	66.3 ± 3.2	66.4 ± 3.0	64.7 ± 4.5	61.3 ± 4.1
Coverage (%)	3.9 ± 0.1	3.8 ± 0.2	**3.2 ± 0.3** ^*^	3.6 ± 0.2	**3.0 ± 0.2** ^*^	3.3 ± 0.2
Cell size (px)	629 ± 17	598 ± 14	627 ± 14	650 ± 7	599 ± 26	**548 ± 20** ^**# +**^
Cell body size (px)	87 ± 4	100 ± 7	87 ± 5	93 ± 2	97 ± 5	98 ± 5
Processes size (px)	554 ± 17	532 ± 22	540 ± 13	541 ± 26	504 ± 26	**470 ± 20** ^**# +**^
**Hilus**						
Density (#/area)	56.8 ± 3.1	56.0 ± 4.4	46.6 ± 2.8	57.1 ± 4.1	**37.9 ± 3.3** ^*^	**48.6 ± 3.1** ^**#**^
Coverage (%)	4.3 ± 0.3	4.6 ± 0.3	3.9 ± 0.3	4.3 ± 0.3	4.5 ± 0.4	4.6 ± 0.3
Cell size (px)	512 ± 21	491 ± 28	545 ± 29	483 ± 26	506 ± 27	482 ± 20
Cell body size (px)	99 ± 5	94 ± 6	106 ± 6	90 ± 5	99 ± 6	94 ± 5
Processes size (px)	420 ± 23	378 ± 13	425 ± 35	388 ± 27	411 ± 24	400 ± 23

ISO, isoproterenol.

Significant effects (p < 0.05) are presented in bold.

* Significant effect of the intervention (WBV or running) compared to sedentary within the same pre-treatment (ISO or saline).

# Significant effect of ISO compared to saline within the same intervention.

+ Significant differences between WBV and running within the same pre-treatment.

#### 3.2.6. Hippocampal BDNF

Brain-derived neurotrophic factor expression was used as a measure of neuronal function (Figures 5B, D, 7). Although overall hippocampal BDNF expression appeared not significantly different between groups (Figure 5D), results presented in Figure 7 suggested regional differences within the hippocampus. Two-way ANOVA showed significant differences in the CA1 area (*p* = 0.030), reflected in a significant effect of interventions (*p* = 0.025), which resulted in ISO causing a significantly increased BDNF expression (*p* = 0.043), that was reversed by running exercise (*p* = 0.003) as well as by WBV (*p* = 0.013) in *post-hoc* testing (Figure 7). Similar effects were observed in the DG and hilus areas; DG: a trend toward differences between groups (*p* = 0.071), resulting in significantly increased expression after ISO (*p* = 0.050), which was reversed by both running exercise (*p* = 0.043) as well as WBV (*p* = 0.005); hilus: significant differences between groups (two-way ANOVA: *p* = 0.020), with a trend toward effects of interventions (*p* = 0.055) and significant interactions between effects of ISO and interventions (*p* = 0.034). ISO significantly increased BDNF expression (*p* = 0.018), which was reversed by running exercise (*p* = 0.003) as well as WBV (*p* = 0.002). In the CA3 area, no significant differences were observed.

No correlations were observed between microglia activity and BDNF expression in the overall hippocampal data, nor in any of the hippocampal areas. Neither microglia activity nor BDNF expressions were significantly associated with collagen expression in the specific hippocampal areas.

#### 3.2.7. Behavior

**Figure 8:**
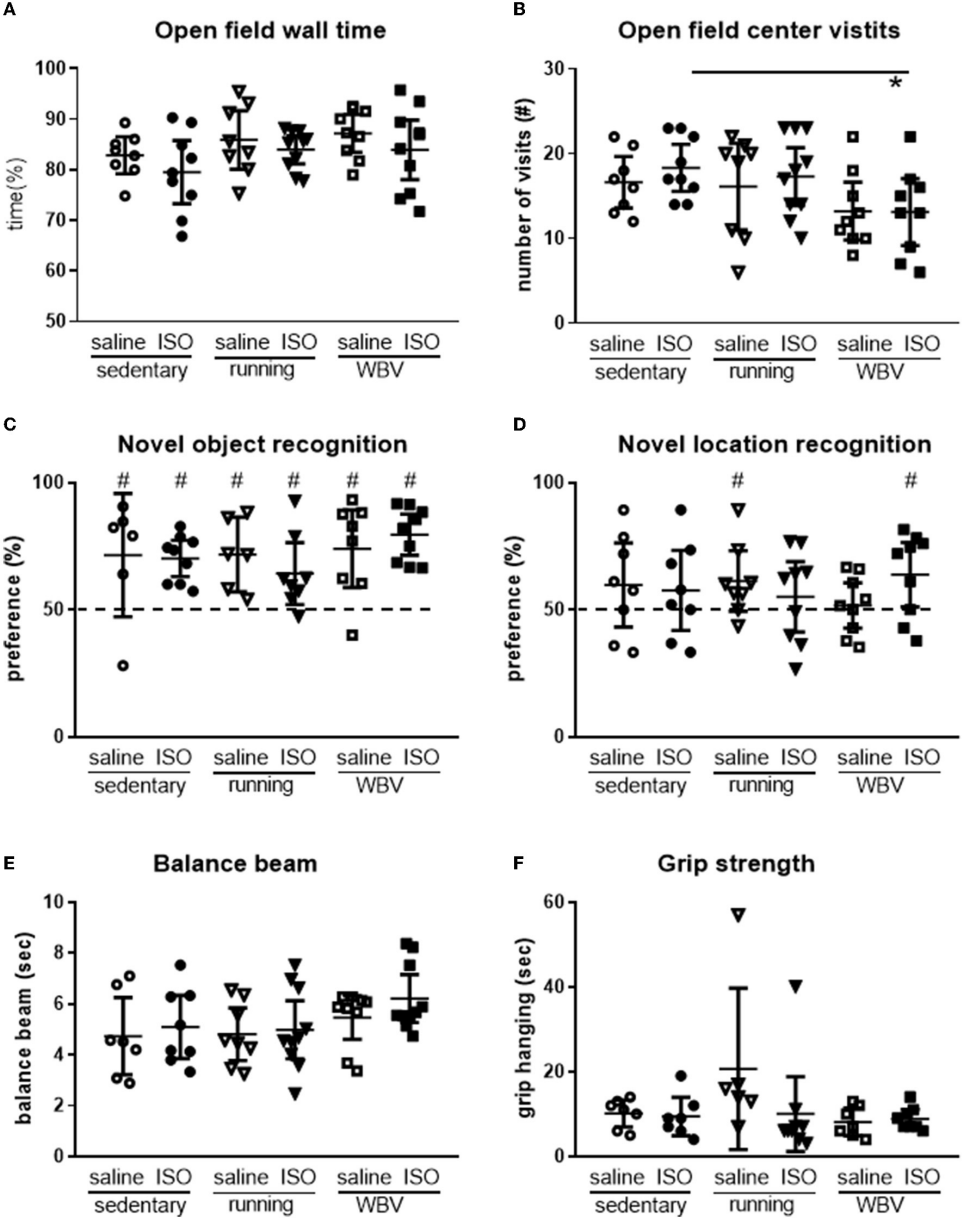
Exploration in the open field (*n* = 8–10 per group) was measured as time spent along the wall **(A)** and the number of center visits **(B)**, cognitive performance measured in the novel object- [**(C)**; *n* = 6–10 per group] and the novel location recognition [**(D)**; *n* = 8–10 per group] tests, and motor performance measured by the time rats needed to cross the balance beam [**(E)**; *n* = 7–10 per group] and the time they could keep their grip on the hanging bar (**F**; *n* = 6–9 per group) in saline- or isoproterenol (ISO)-treated rats, under sedentary conditions, or after 5 weeks of exercise training (running) or whole-body vibration (vibration). *Significant difference between indicated groups (*p* < 0.05); ^#^significantly above chance level (dashed line = 50% = chance level).

Levels of exploration and anxiety were obtained from behavior in the OF. Regarding exploration, a tendency for differences in locomotion activity between the groups was observed (*p* = 0.07), resulting from a significant effect of interventions (*p* = 0.012). *Post-hoc* analyses indicated that WBV rats displayed reduced locomotion (sedentary saline: 26 ± 2; sedentary ISO: 28 ± 2; sedentary running: 25 ± 4; running ISO: 27 ± 2; sedentary WBV: 19 ± 2; WBV ISO: 21 ± 3 crossings per 5 min). No differences between groups, regarding ISO/saline, interventions, or interactions, were observed for the time rats spent in the relatively safe area and the wall. For the number of center visits, ANOVA revealed a tendency for differences between groups (*p* = 0.10), which could be attributed to a significant effect of the intervention (*p* = 0.020), as no effect of ISO vs. saline nor interactions were observed. ISO rats with WBV showed a reduction in center visits (*p* = 0.025; Figures 8A, B). Effects on cognition were measured as short-term memory in the NOR and NLR tests (Figures 8C, D). All rat groups could recognize the novel object in the NOR, as they all performed significantly above the chance level. However, no difference in performance between the groups was observed. Similarly, in the NLR, no differences between groups were seen, but here only saline runners and WBV ISO rats performed above the chance level. Motor performance was obtained from the balance beam test and the grip hanging test, presented in Figures 8E, F. Balance beam performance appeared similar in all groups. In the grip hanging test, the saline-treated runners stood out, as they seem to perform better than all other groups. No significant associations between behavioral parameters and microglia activity or BDNF expression were observed.

## 4. Discussion

### 4.1. General

Myocardial infarction is often associated with mental disorders, including depression and cognitive decline, with female patients being more susceptible than male patients. Exercise training may provide a rational therapeutic approach for this comorbidity, due to its known beneficial effects on physical as well as mental conditions. However, physical exercise may not be appreciated shortly after myocardial infarction. WBV could provide a passive alternative to exercise in this condition (Alam et al., 2018). The present study aimed to explore WBV compared to physical exercise in female rats after ISO-induced myocardial damage. Exercise, but not WBV, reversed cardiac damage. Surprisingly, collagen was also expressed in the hippocampus and a reduced expression was found after exercise as well as after WBV in the hippocampal CA3 of ISO-treated rats. Effects of ISO on microglia activity varied from increased (CA1), no difference (DG and hilus), to decreased (CA3). Both exercise and WBV normalized the effects of ISO. The ISO-induced elevated BDNF expression was no longer present after exercise or WBV. These WBV effects were associated with less locomotion and lower interest in the center area of the OF, but preserved short-term spatial memory in ISO-treated rats; an effect that was not observed after exercise. On the contrary, exercise, but not WBV, seemed to improve grip strength. Results indicate that apart from the lack of effect on cardiac collagen, the effects of WBV appeared quite comparable to exercise, indicating that WBV may provide a valuable alternative for patients who cannot perform physical exercise. However, more research on the optimal WBV protocol is necessary.

### 4.2. WBV vs. exercise

#### 4.2.1. General

In the present study, WBV was evaluated as an alternative to physical exercise after myocardial damage, as WBV and physical exercise share effects on the body, such as improved muscle strength (Annino et al., 2017, 2021; Beaudart et al., 2017), bone density (Slatkovska et al., 2010; Benedetti et al., 2018), and wound healing (Zhou et al., 2016; Wano et al., 2021), as well as on the brain, including neurotrophic factors (Raval et al., 2018; Mee-Inta et al., 2019) and cognitive improvement (Keijser et al., 2017; Boerema et al., 2018; Cardoso et al., 2022). As reviewed by Alam et al. (2018), WBV is regarded as a neuromuscular training method, that could be used as an alternative to conventional training. Moreover, WBV was shown to reduce brain damage and brain inflammatory markers, with increased BDNF and improved functional activity after transient brain ischemia in middle-aged female rats (Raval et al., 2018).

#### 4.2.2. Effects on the heart

As anticipated from our previous study in female rats (Toth et al., 2022a), ISO increased cardiac collagen levels. Although exercise by itself may slightly increase cardiac collagen in saline-treated rats, potentially by increasing fibroblast growth factor 21 (Ma et al., 2021), the ISO-induced cardiac fibrosis was completely reversed. WBV, however, seemed to even exaggerate ISO-induced cardiac fibrosis. In contrast, WBV was reported to increase tolerance to ischemia-reperfusion injury by reducing infarct size in rats (Shekarforoush and Naghii, 2019). On the contrary, in patients, although the significant improvement was seen after the standard exercise rehabilitation program, no extra effects of WBV were observed (Nowak-Lis et al., 2022). Both studies, however, were performed only on male subjects. In our previous studies, we showed different responses to ISO in male and female rats, whereas in male rats, ISO increased cardiac collagen, but no effect of exercise was observed (Toth et al., 2021), in female rats of the same age, exercise significantly reduced the ISO-induced cardiac fibrosis (Toth et al., 2022a). Hence, in our middle-aged female rats, exercise, but not WBV, was capable of reversing cardiac damage due to ISO treatment.

#### 4.2.3. Effects on the brain

##### 4.2.3.1. Hippocampal collagen

In addition to the expected expression in brain blood vessel walls and meninges, collagen expression was observed in the granular layers of the hippocampus, where it may reflect extracellular matrix components. The presence of a neuronal cell surface feature, called perineuronal net (PNN), is consistent with a brain extracellular matrix (Bonneh-Barkay and Wiley, 2009). This PNN mainly covers the cell body and dendrites, is usually associated with neuroprotection, and plays an important role in learning, memory, and information processing in health and disease (Krishnawamy et al., 2021). More specifically, it may affect synaptic morphology and function. Loss of PNN is often observed in neurodegenerative diseases, as reviewed by Bonneh-Barkay and Wiley (Bonneh-Barkay and Wiley, 2009). Moreover, PNNs around hippocampal interneurons can resist destruction by activated microglia (Schuppel et al., 2002).

Although WBV often has been associated with increased collagen in the peripheral body, to the best of our knowledge, no literature is available for effects on brain (hippocampal) collagen. In the present study, no effects of ISO treatment or subsequent intervention with running or WBV were observed in the hippocampal CA1, DG, and hilus areas. However, although not statistically significant, WBV caused a consistent increase in collagen expression in the CA2 in saline-treated rats, but not in ISO-treated rats. In the hippocampal CA2 area, the PNN is known to play a role in restricting synaptic plasticity (Carstens et al., 2016). In the CA3 area, running as well as WBV decreased collagen expression in ISO treated rats. If indeed collagen levels might reflect PNN and restrict synaptic plasticity, as described for the CA2 (Carstens et al., 2016), the reduction of collagen by exercise and WBV in ISO-treated rats may then be speculated as an improvement of synaptic plasticity. Alternatively, it may still point to the loss of neuroprotection (Krishnawamy et al., 2021).

##### 4.2.3.2. Neuroinflammation

Exercise (Petersen and Pedersen, 2005) and WBV (Jawed et al., 2020; Sanni et al., 2022) are associated with anti-inflammatory effects. This anti-inflammatory property may extend to neuroinflammation (Mee-Inta et al., 2019; Chen et al., 2022; Oroszi et al., 2022a). Based on the literature, WBV may affect neuroinflammation in female rats (Raval et al., 2018) as well as in male rats (Oroszi et al., 2022a). However, neither in male (Toth et al., 2021) nor in female rats (Toth et al., 2022a), exercise affected microglia activity after ISO. In the present study, overall hippocampal microglia activity was neither affected by ISO, nor by the interventions. However, the areas within the hippocampus showed regional differences; whereas in the CA1, indeed microglia activation was observed after ISO, and was completely reversed by exercise as well as WBV, in the CA3 area, ISO caused a decline of microglia activity, which was also completely reversed by exercise and WBV. While both the CA1 and CA3 areas are involved in learning and memory (Stevenson et al., 2020), the CA3 area is rather specifically involved in pattern completion (Stevenson et al., 2020). We did not perform behavioral testing to specifically explore the potential role of the CA3. For the CA1, the outcome of the NLR was not found to correlate with microglia activity. Therefore, a direct relationship between neuroinflammation and behavior could not be established here. Alternatively, different time courses for the different parameters, as seen before (Hovens et al., 2014b), may provide a potential explanation for the observed differences in effects in CA1 and CA3.

##### 4.2.3.3. Neuronal function (BDNF)

Exercise training is usually associated with increased brain BDNF expression (Sleiman et al., 2016; El Hayek et al., 2019). Exercise-induced increased expression of BDNF and double cortin positive cells were observed in the ischemic hippocampus after stroke in rats (Luo et al., 2019; Cheng et al., 2020). WBV was shown to increase BDNF levels in the peri-infarct regions after brain ischemia-reperfusion in middle-aged female rats (Raval et al., 2018). Furthermore, WBV was demonstrated to reverse the decreased level of BDNF in the CA1 area of the hippocampus induced by chronic restrain stress in male rats (Peng et al., 2021). In male rats, ISO had no effects on hippocampal BDNF expression 6 weeks later, but running exercise significantly increased BDNF expression in the CA1 and hilus areas of the hippocampus (Toth et al., 2021). In contrast, in female rats, exercise after ISO seemed to decline BDNF expression in the CA1 area (Toth et al., 2022a). Accordingly, in the present study, the ISO-induced increases in BDNF in the sedentary rats were completely reversed by exercise as well as by WBV. Since most of the results of exercise-induced increases in BDNF expression were obtained in male subjects, the deviant results in female rats in the present study may well be attributed to sex dimorphism (Toth et al., 2022b).

#### 4.2.4. Effects on behavior

##### 4.2.4.1. Depression/anxiety

Although we anticipated cardiac damage-induced anxiety/depressive-like behavior in the ISO-treated rats (Tkachenko et al., 2018; Hu et al., 2020), in agreement with our previous study on middle-aged female rats (Toth et al., 2022a), ISO with and without exercise training had no effect on behavior in the OF. Similarly, at 24 months of age, no effect of ISO was observed in OF behavior in female rats, but anxiety-associated effects were seen in male rats (Toth et al., 2022b). However, although the lower number of center visits of the ISO-treated WBV rats in the present study may point to a higher level of anxiety after WBV, no effect on wall time, as a measure for depressive-like behavior, was observed. The isolated reduction of center visits may therefore rather reflect the previously described reduction of arousal after WBV (Boerema et al., 2018), which would be further supported by the reduced OF locomotion of these rats (Oroszi et al., 2022a). Taken together, the results of the different studies indicated that neither ISO nor exercise affected OF behavior in female rats, but the effects of WBV on OF behavior remained inconclusive.

##### 4.2.4.2. Cognition

The pilot exposure time–effect study did not show significant effects of either WBV schedule on short-term memory in the NOR and NLR tests. Similarly, in the main study, no effect of WBV (nor exercise) was observed in the results of the NOR. However, similar to our previous study on middle-aged female rats (Toth et al., 2022a), saline-treated exercise rats performed above chance level in the NLR, an effect that was not seen in the saline WBV rats. Whereas, exercise could not overcome poor performance in rats after ISO, WBV after ISO seemed to improve performance to above chance level.

##### 4.2.4.3. Motor performance

No effects of ISO were observed on motor performance, tested either on the balance beam or in the grip hanging test. Exercise is generally accepted to improve motor performance (Hubner and Voelcker-Rehage, 2017) but actual effects may depend on the exercise protocol. Exercise by itself seemed to improve grip hanging, with large variation, however, but had no effect after ISO. Muscle weight was not increased by either exercise or WBV, indicating no significant effect of training. Several literature studies indicate a positive effect of WBV on muscle strength and motor coordination, although often in combination with regular physical training (Kawanabe et al., 2007; Annino et al., 2017, 2021). In the present study, no significant effect of WBV was seen on balance beam performance. Accordingly, in our pilot exposure time–effect study, no significant effects were observed on motor performance. However, 5 min of WBV already seemed to double grip hanging, without further increase with longer WBV exposure times. Studies in male mice showed that 5 min, but not 30 min, daily WBV improved motor performance (Keijser et al., 2017), and 5 min, but not 20 min, WBV improved rearing in the OF and grip hanging in male rats (Oroszi et al., 2022a). Moreover, in a recent study of our group in aged male and female rats (Oroszi et al., 2022b), behavioral effects of 10 min daily WBV appeared rather mild in female rats compared to male rats. Therefore, apart from male mice vs. female rats, the effects of WBV on motor performance may largely depend on the treatment protocol.

### 4.3. Limitations

The ISO model was used to avoid surgery-induced brain and behavioral changes that were observed after coronary artery ligation (Hovens et al., 2016). However, we are well aware that this provides a model for the consequences of cardiac damage, rather than its etiology of it. To compare the effects of WBV to exercise after ISO, the exercise protocol was based on our previous studies in male and female rats (Toth et al., 2021, 2022a) and the WBV protocol on our exposure time response pilot. Cardiac function measurements in the female rat study suggested a reduced cardiac performance after exercise, which may suggest a too-severe exercise protocol for these females. It raises the question of what parameter(s) should be optimal for the comparison of the two rather different interventions. As discussed in our previous study in female rats (Toth et al., 2022a), interventions started 1 week after ISO treatment, when inflammatory processes are merely complete (Alemasi et al., 2019). Accordingly, ISO induced cardiac damage, but that did not result in major effects on behavior 6 weeks later, leaving limited scope for improvement by either exercise or WBV. Exercise, but not WBV, reversed cardiac damage, but indeed neither did affect behavior. Nevertheless, in the present study, clear effects on local brain parameters were observed, providing a new potential entrée for treatment, specifically regarding the CA3 area and its associated functions, as this area stood out in the measured brain parameters.

## 5. Conclusion

The study aimed to explore WBV after ISO as an alternative to exercise, on the heart, the brain, and behavior in female rats. Although the female rats did not show the anticipated depressive-like behavior or cognitive decline after ISO, our data indicated regional effects on neuroinflammation and BDNF expression in the hippocampus, which were merely normalized by both WBV and exercise. Furthermore, collagen expression was observed in the granular layers of the hippocampus and appeared regionally specific and sensitive to exercise as well as WBV in ISO-treated rats. Therefore, apart from the potential concern about the lack of cardiac collagen reduction, WBV may provide a relevant alternative to physical exercise and be of help to (female) subjects that cannot or are not motivated to perform the exercise.

## Data availability statement

The original contributions presented in the study are included in the article/supplementary material, further inquiries can be directed to the corresponding author.

## Ethics statement

The experiments were conducted under the general license for animal experiments of the Laboratory of Physical Education, University of Budapest, Hungary.

## Author contributions

KT: design of the study, acquisition of data, analyses and interpretation of data, and substantively revised the manuscript. TO: performing behavioral studies, acquisition of data, and substantive revision of the manuscript. CN: conception, design of the study, and interpretation of data. EZ: interpretation of data and substantively revised the manuscript. RS: conception, design of the study, analyses and interpretation of data, drafting of the manuscript, and substantive revision of the manuscript. All authors contributed to the article and approved the submitted version.
